# The effects of focal adhesion kinase and platelet-derived growth factor receptor beta inhibition in a patient-derived xenograft model of primary and metastatic Wilms tumor

**DOI:** 10.18632/oncotarget.27165

**Published:** 2019-09-17

**Authors:** Jamie M. Aye, Laura L. Stafman, Adele P. Williams, Evan F. Garner, Jerry E. Stewart, Joshua C. Anderson, Smitha Mruthyunjayappa, Mary G. Waldrop, Caroline D. Goolsby, Hooper R. Markert, Colin Quinn, Raoud Marayati, Elizabeth Mroczek-Musulman, Christopher D. Willey, Karina J. Yoon, Kimberly F. Whelan, Elizabeth A. Beierle

**Affiliations:** ^1^Department of Pediatrics, Division of Hematology Oncology, University of Alabama at Birmingham, Birmingham, AL, USA; ^2^Department of Surgery, University of Alabama at Birmingham, Birmingham, AL, USA; ^3^Department of Radiation Oncology, University of Alabama at Birmingham, Birmingham, AL, USA; ^4^Department of Pathology, University of Alabama at Birmingham, Birmingham, AL, USA; ^5^Department of Pharmacology and Toxicology, University of Alabama at Birmingham, Birmingham, AL, USA

**Keywords:** kinase inhibition, Wilms tumor

## Abstract

Aggressive therapies for patients with metastatic Wilms tumor (WT) with subsequent severe late effects warrant the search for novel therapies. The role of focal adhesion kinase (FAK), a non-receptor tyrosine kinase important in pediatric solid tumor development and progression, has not been examined in metastatic WT. Using a novel patient-derived xenograft (PDX) of a primary and matched, isogenic, metastatic WT, the hypothesis of the current study was that FAK would contribute to metastatic WT and small molecule inhibition would decrease tumor growth. Immunohistochemical staining, immunoblotting, cell viability and proliferation assays, cell cycle analysis, and cellular motility and attachment-independent growth assays were performed. FAK was present and phosphorylated in both WT PDXs and in the human samples from which they were derived. FAK inhibition decreased cellular survival, proliferation, and cell cycle progression in both PDXs but only significantly decreased migration, invasion, and attachment-independent growth in the primary WT PDX. Kinomic profiling revealed that platelet-derived growth factor receptor beta (PDGFRβ) may be affected by FAK inhibition in WT. Pharmacologic inhibition of FAK and PDGFRβ was synergistic in primary WT PDX cells. These findings broaden the knowledge of metastatic WT and support further investigations on the potential use of FAK and PDGFRβ inhibitors.

## INTRODUCTION

Wilms tumor (WT) is the most common primary pediatric renal malignancy. Approximately 12% of patients with WT will have metastatic disease at diagnosis [1]. The 4-year-relapse-free survival rate for metastatic, favorable histology WT is approximately 70% [2]. Patients with unfavorable histology and metastatic disease at diagnosis have a graver prognosis with an overall 4-year survival rate of only 33% [2]. The most common sites of metastases are the lung (80%) and the liver (15%) [3]. Current therapy for metastatic WT involves a multimodal, aggressive approach with surgery, radiation, and chemotherapy, and 25% of patients experience severe chronic late effects warranting investigation of novel therapies [2].

Focal adhesion kinase (FAK) is a 125 kDa non-receptor tyrosine kinase involved in cell adhesion, migration, invasion, proliferation, and survival [4]. FAK is activated by the binding of cell surface integrins and auto-phosphorylation at tyrosine 397 (Y397 FAK). FAK phosphorylates and activates the Src family of kinases which affects downstream pathways, such as the activation of the phosphatidylinositide 3′-OH-kinase-Akt pathway and the inhibition of the caspase-3 cascade, leading to inhibition of apoptosis [5].

Proper formation of nephrons in the developing kidney involves the activation of FAK to stimulate coordinated extension of cell processes and cell migration of the ureteric bud epithelium through the blastemal matrix [6]. Normal postnatal kidneys demonstrate FAK Y397 localization to the basolateral cell membrane at the interface between collecting tubule epithelia and the extracellular matrix [7] and FAK signaling is required for collecting duct branching [8]. FAK Y397 and FAK expression declines with renal maturity [9, 10], and early pharmacologic inhibition of FAK inhibits normal nephron formation *in vitro* [8].

In the same way FAK is involved in the invasive behavior of normal renal development, FAK signaling is thought to be required for the invasion of neoplastic cells [8]. Early studies of FAK in normal tissue compared to primary and metastatic colon carcinomas from individual patients demonstrated a progressive increase in mRNA levels suggesting FAK confers metastatic potential [11]. Several studies have since demonstrated overexpression of FAK in a variety of cancer types and significant correlations with tumor size, higher disease stage, and poorer patient prognosis [12]. Migration, adhesion, and invasion are essential for the formation of metastases and inhibition of FAK activity has been shown to decrease these prerequisites for metastases in renal cell carcinoma both *in vitro* [13] and *in vivo* [14]. FAK inhibition has also decreased tumorigenicity in other adult cancers including non-small cell lung cancer, gastric cancer, hepatocellular carcinoma, and bladder cancer [15–18] and in pediatric malignancies including neuroblastoma and Ewing sarcoma [19, 20]. In pediatric renal tumors, FAK inhibition decreased cell viability, migration, and invasion *in vitro* and tumor volume *in vivo* in a malignant rhabdoid kidney tumor cell line [21]. While the specific mechanisms remain to be elucidated, evidence supports that FAK contributes to both tumor formation and malignant progression [22] and these findings formed the rationale for our investigation of FAK in WT.

Kinomic profiling is a new, high-throughput method used to investigate kinase signaling to identify potential therapeutic targets. The PamGene PamChip^®^ system allows direct recording of cellular kinase activity for comparison of phosphorylation of tyrosine or serine/threonine peptides as they are phosphorylated by cellular kinases [23]. This system has been used to profile a variety of malignancies including renal cell carcinoma [24].

Currently there are only a limited number of cell lines available for the study of metastatic WT, such as WiT49 and CCG-99-11 [25]. We established a novel patient-derived xenograft (PDX) model of a liver metastasis, COA 42, and a PDX of its matched isogenic primary renal WT, COA 25, to investigate the roles of FAK in WT. Because FAK is only one of many kinases involved in tumorigenesis, we also sought to explore kinases upstream and downstream of FAK. We hypothesized that FAK plays a role in the tumorigenicity of metastatic WT and that FAK inhibition would result in a less aggressive phenotype in metastatic WT.

In the current study, we demonstrated abrogation of FAK in PDX cell lines of primary and metastatic WT resulted in decreased tumorigenicity *in vitro*. Additionally, with kinomic profiling, we discovered other pathways affected by FAK inhibition in WT including PDGFRβ. We then investigated inhibition of both FAK and PDGFRβ and demonstrated that dual inhibition synergistically decreased primary WT cell survival. While Phase I studies of FAK inhibitors in adults with recurrent or metastatic solid tumors have demonstrated tolerability and stabilization of disease [26, 27], no Phase I studies of FAK inhibitors in children with recurrent or metastatic solid tumors, including WT, have been performed. Overall, the current study is the first to examine the role of FAK in WT and findings support further preclinical investigations on the potential use of FAK inhibitors either alone or in combination with other kinase inhibitors in the treatment of WT.

## RESULTS

### Both primary and metastatic WT expressed FAK

**Figure 1:**
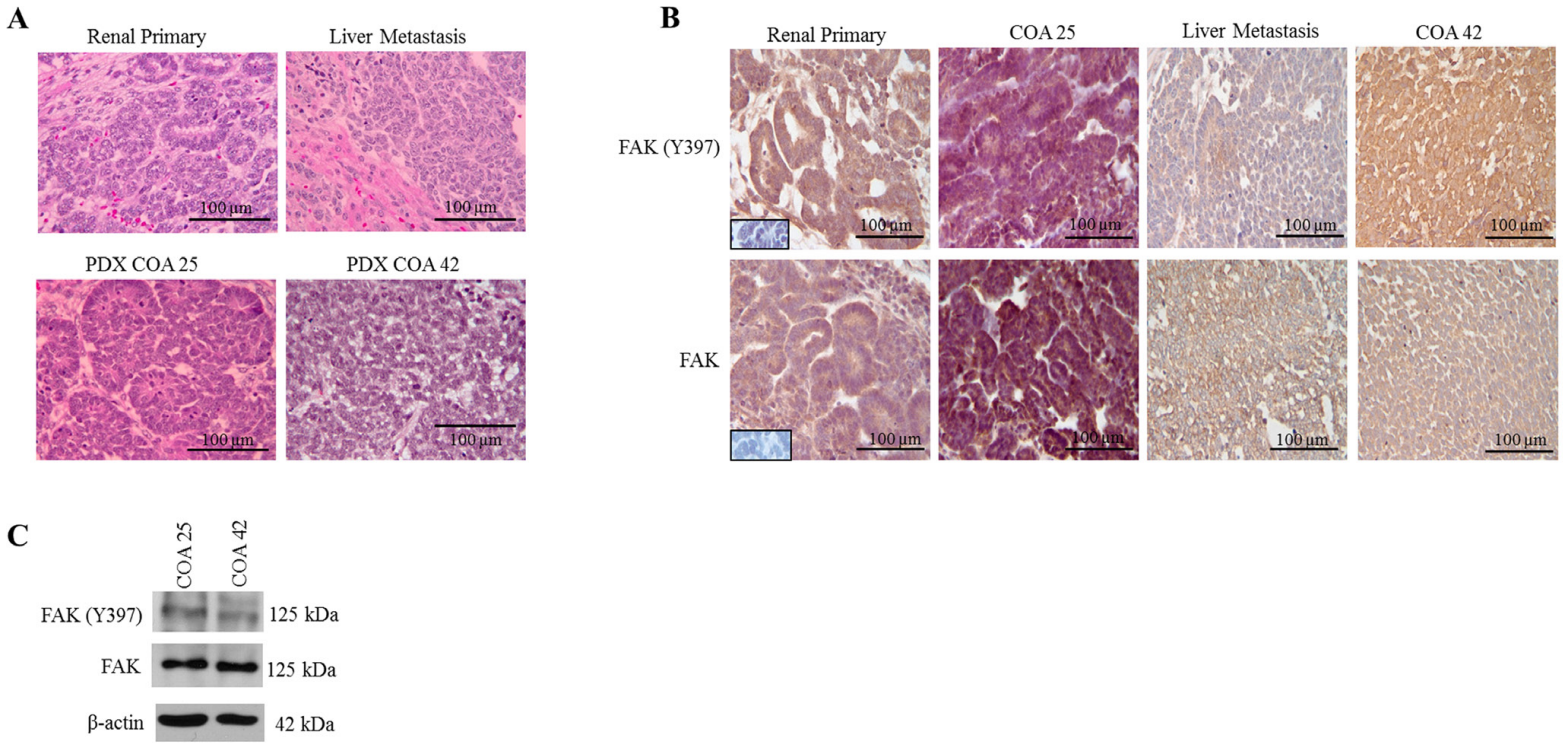
FAK in human Wilms tumor (WT) samples and patient derived xenografts (PDX). (**A**) H&E staining demonstrating PDX COA 25 (*left lower panel*) recapitulated the predominant epithelial histology of the patient’s primary renal WT (*left upper panel*) and PDX COA 42 (*right lower panel*) recapitulated the predominant blastemal histology of the patient’s liver metastasis (*right upper panel*). (**B**) Immunohistochemistry staining with antibodies for FAK Y397 and total FAK was performed on the primary renal tumor, liver metastasis, and PDXs - COA 25 and COA 42. Negative controls were included for each sample (*inserts*). Staining for FAK Y397 and total FAK was positive in all samples and located in tumor cell cytoplasm and membrane. (**C**) Immunoblotting for FAK Y397 and total FAK was performed on COA 25 and COA 42 cell lysates. Y397 FAK and total FAK were detected in both PDXs.

H&E staining of human primary renal WT, liver metastasis, and COA 25 (primary) and COA 42 (metastasis) PDXs was performed. Staining confirmed PDX COA 25 (Figure 1A, *lower left panel*) recapitulated the patient’s primary renal WT (Figure 1A, *upper left panel*) with both consisting primarily of epithelial cells. Additionally, PDX COA 42 (Figure 1A, *lower right panel*) recapitulated the patient’s liver metastasis (Figure 1A, *upper right panel*) with both consisting primarily of blastemal cells. Immunohistochemical staining for phosphorylated FAK (FAK Y397) and total FAK was performed on human primary and metastatic samples as well as PDXs COA 25 and COA 42. Staining demonstrated phosphorylated FAK and total FAK expression was present in the epithelial cells of the human primary renal WT and COA 25 (Figure 1B, *left panels*) and in the blastemal cells of the metastatic WT and COA 42 (Figure 1B, *right panels*). The subcellular localization of FAK and FAK Y397 was located in the tumor cell cytoplasm and membrane of all samples. Both PDXs expressed phosphorylated FAK and FAK by immunoblotting of cell lysates (Figure 1C).

### FAK inhibition with PF and Y15 decreased cell survival and proliferation

**Figure 2:**
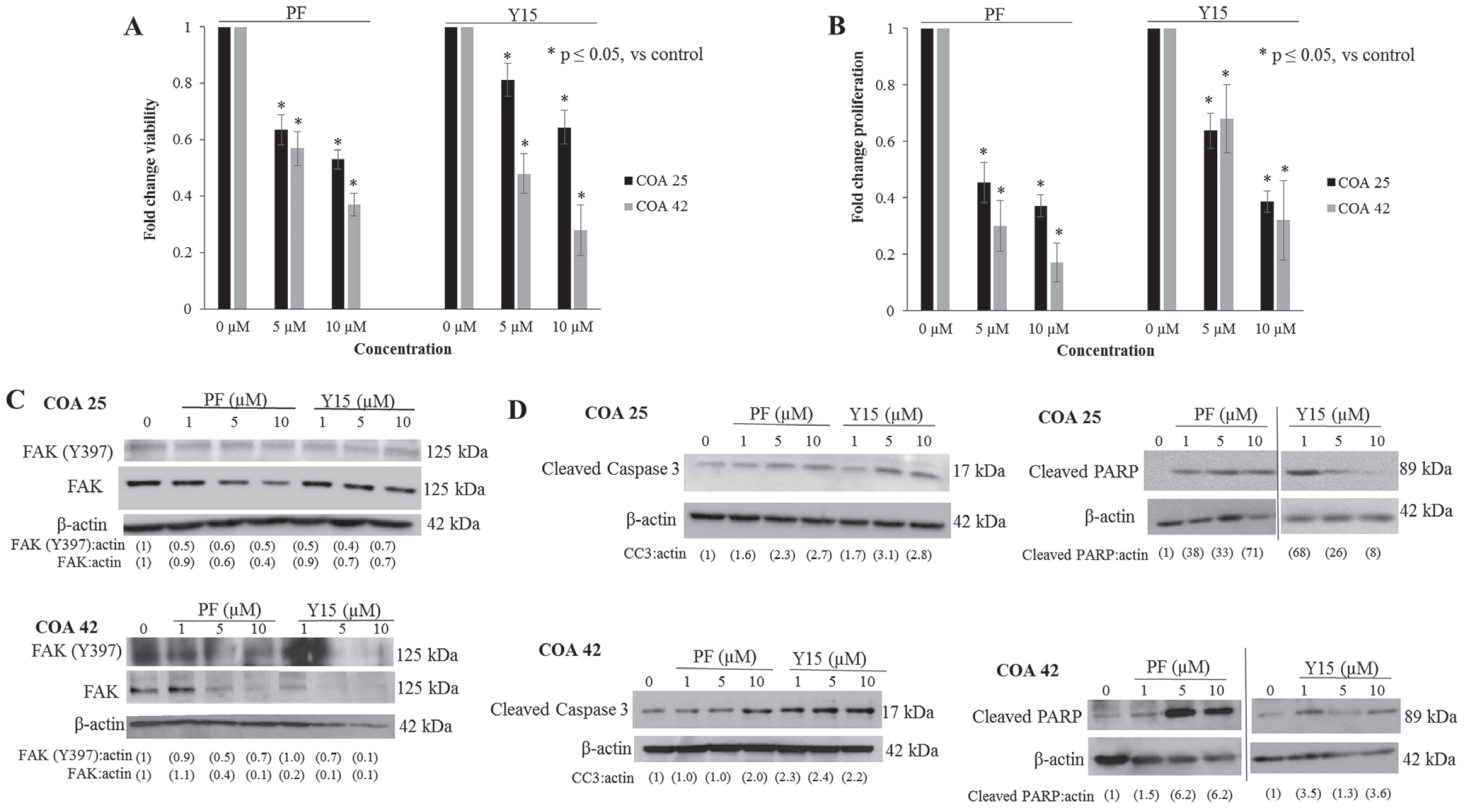
PF-573,228 (PF) and 1,2,4,5-benzenetetraamine tetrahydrochloride (Y15) inhibition of FAK decreased cell survival and proliferation and increased apoptosis. (**A**) COA 25 and COA 42 cells (6 × 10^4^/well) were treated for 24 hours with increasing concentrations of PF or Y15. AlamarBlue^®^ assays were used to assess cell survival. Both COA 25 and COA 42 showed significantly decreased cell survival following treatment with PF and Y15 for 24 hours. (**B**) COA 25 and COA 42 cells (1.5 × 10^4^/well) were treated for 24 hours with increasing concentrations of PF or Y15. CellTiter 96^®^ assays were used to assess cell proliferation. Both COA 25 and COA 42 showed significantly decreased proliferation following treatment with PF and Y15 for 24 hours. (**C**) COA 25 and COA 42 cells were treated for 24 hours with increasing concentrations of PF or Y15. Cell lysates were harvested and evaluated with immunoblotting for FAK Y397 and total FAK. Increasing concentrations of PF and Y15 decreased FAK Y397 and total FAK in both cell lines. (**D**) COA 25 and COA 42 cells were treated for 24 hours with increasing concentrations of PF or Y15. Immunoblotting for cleaved caspase 3 and cleaved PARP were used to detect apoptosis. Immunoblotting demonstrated an increase in cleaved caspase 3 and cleaved PARP with increasing concentrations of PF and Y15 in COA 25 and COA 42 cells, demonstrating apoptosis. Data represent mean ± SEM of three biologic replicates.

AlamarBlue^®^ assays were used to determine the effects of FAK inhibition on cell survival. Cells were treated with PF or Y15 for 24 hours at increasing concentrations. Cell survival was significantly decreased in both PDXs following treatment with PF and Y15 (Figure 2A). The calculated LD_50_ for PF in COA 25 cells was 9.8 µM and in COA 42 cells was 7.2 µM. The calculated LD_50_ for Y15 was 14.5 µM in COA 25 cells and 6.1 µM in COA 42 cells. The LD_50_ for HEK 293 cells for PF and Y15 were 29.8 µM and 28.4 µM, respectively. Additionally, using CellTiter 96^®^ assays, treatment with PF and Y15 was found to significantly decrease cell proliferation in both COA 25 and COA 42 PDXs (Figure 2B). Immunoblotting was used to confirm FAK abrogation and demonstrated PF and Y15 decreased FAK expression and subsequent phosphorylation in both cell lines (Figure 2C).

FAK inhibition has been shown to lead to apoptosis [18]. To determine whether decreased viability was due to apoptosis, cell lysates were examined for cleavage products of caspase 3 and PARP. There was an increase in cleaved caspase 3 (Figure 2D, *upper left panel*) and cleaved PARP (Figure 2D, *upper right panel*) in COA 25 cells treated with PF and Y15 indicating apoptosis. In COA 42 cells, cleaved caspase 3 (Figure 2D, *lower left panel*) and cleaved PARP (Figure 2D, *lower right panel*) increased with PF and Y15 treatment indicating apoptosis following PF or Y15 treatment.

### FAK inhibition decreased cell migration, invasion, and attachment-independent growth

**Figure 3:**
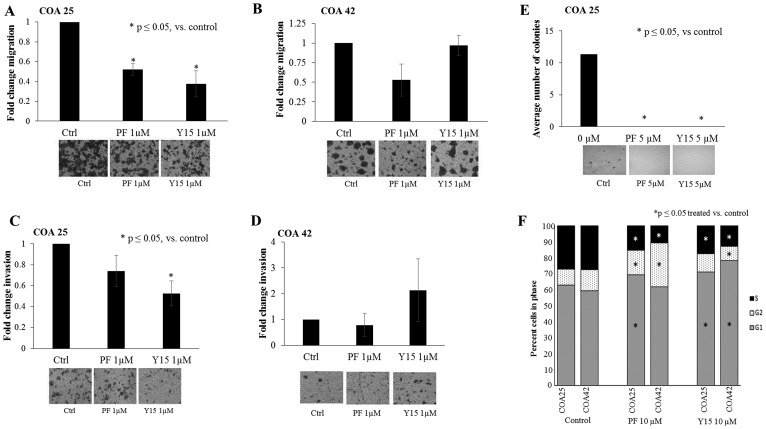
PF-573,228 (PF) and 1,2,4,5-benzenetetraamine tetrahydrochloride (Y15) inhibition of FAK decreased cell migration, invasion, and attachment-independent growth in COA 25 cells. (**A**) COA 25 cells (5 × 10^5^) were treated for 24 hours with PF (1 µM) or Y15 (1 µM) and allowed to migrate through a micropore insert. Migration was reported as fold change in the percentage of area migrated. Migration significantly decreased with PF and Y15 treatment. (**B**) COA 42 cells (5 × 10^5^) were treated for 24 hours with PF (1 µM) or Y15 (1 µM) and allowed to migrate through a micropore insert. Migration was reported as fold change in the percentage of area migrated. Migration did not significantly decrease with PF or Y15 treatment. (**C**) COA 25 cells (5 × 10^5^) were treated for 24 hours with PF (1 µM) or Y15 (1 µM) and allowed to invade through a Matrigel™-coated micropore insert. Invasion was reported as fold change in the percentage of area invaded. Invasion significantly decreased with Y15 treatment. (**D**) COA 42 cells (5 × 10^5^) were treated for 24 hours with PF (1 µM) or Y15 (1 µM) and allowed to invade through a Matrigel™-coated micropore insert. Invasion was reported as the fold change in percentage of area invaded. Invasion did not significantly decrease with PF or Y15 treatment. (**E**) Attachment-independent growth in soft agar was used to characterize tumor invasiveness in the COA 25 cells. Cells (3 × 10^5^/dish) were treated with PF (5 µM) or Y15 (5 µM) for 8 weeks and colonies were quantified using Image J. The average number of colonies significantly decreased after treatment with PF and Y15. Representative photographs of migration, invasion, and soft agar plates are situated below each graph. (**F**) COA 25 and COA 42 cells (1 × 10^6^) were treated for 24 hours with PF or Y15. Cell cycle analysis demonstrated a significantly increased percentage of cells in G_1_ and a decreased percentage in S phase for COA 25 and COA 42 cells treated with PF and Y15 (10 µM), indicating a lack of progression through the cell cycle. All experiments were repeated with at least three biologic replicates and data reported as mean ± SEM.

FAK has been shown to play a crucial role in the successful metastasis of tumor cells [28], leading to the investigation of FAK inhibition on migration and invasion in these PDXs. COA 25 and COA 42 cells (5 × 10^5^) were treated with PF (0, 1 µM) or Y15 (0, 1 µM) and allowed to migrate through a micropore membrane for 24 hours. There was a significant decrease in migration in PF and Y15 treated COA 25 cells (Figure 3A) at concentrations of PF and Y15 that were below the calculated LD_50_. Meanwhile, there was not a significant decrease in migration in COA 42 cells with PF or Y15 (Figure 3B). Similarly, for invasion, (5 × 10^5^) cells were treated with PF or Y15 and allowed to invade through a Matrigel™ layer. While there were decreases in invasion with PF and Y15 treated COA 25 cells, only cells treated with Y15 had a statistically significant decrease in cellular invasion (Figure 3C). Treatment with PF and Y15 did not lead to a change in invasion for COA 42 cells (Figure 3D).

Attachment-independent growth using soft agar is another method to measure the invasive phenotype of cells. Attachment-independent growth was measured following PF and Y15-induced FAK inhibition. COA 25 cells (3 × 10^5^) were treated with PF or Y15, placed into soft agar, and colonies were allowed to grow for 8 weeks. The number of colonies detected at the end of the study period decreased by 100% at concentrations of PF and Y15 (5 µM) below the calculated LD_50_ in COA 25 cells (Figure 3E). COA 42 cells did not grow in attachment-independent conditions.

### FAK inhibition decreased cell cycle progression

Since FAK is known to support progression through the cell cycle [29], cell cycle analysis was investigated. Treatment of COA 25 and COA 42 cells with PF (0, 10 µM) or Y15 (0, 10 µM) resulted in an increased percentage of cells in G_1_ and a decreased percentage in S phase (Figure 3F), indicating a lack of progression through the cell cycle. Representative histograms are presented in Supplementary Figure 1, and data in tabular form in Supplementary Table 1.

### Kinomic alterations of primary and metastatic WT by FAK inhibition

Because FAK is only one of many kinases involved in tumorigenesis, kinomic profiling was used as a hypothesis-generating tool to examine potential therapeutic targets upstream and downstream of FAK. Relative to COA 42, COA 25 cells had increased EPHA8 and ROR1 and decreased PDGFRβ activity at baseline (Supplementary Figure 2A). Twenty-four-hour treatment with PF increased PDGFRβ, RON, P70S6KB, and MAK activity in COA 25 cells (Supplementary Figure 2B). COA 42 cells displayed decreased TNK1, LMR1, CCK4, EPHA5, PDK1, SGK196, LKB1 and increased PSKH1 activity with PF treatment (Supplementary Figure 2C).

### Effects of FAK inhibition on PDGFRβ in primary and metastatic WT

**Figure 4:**
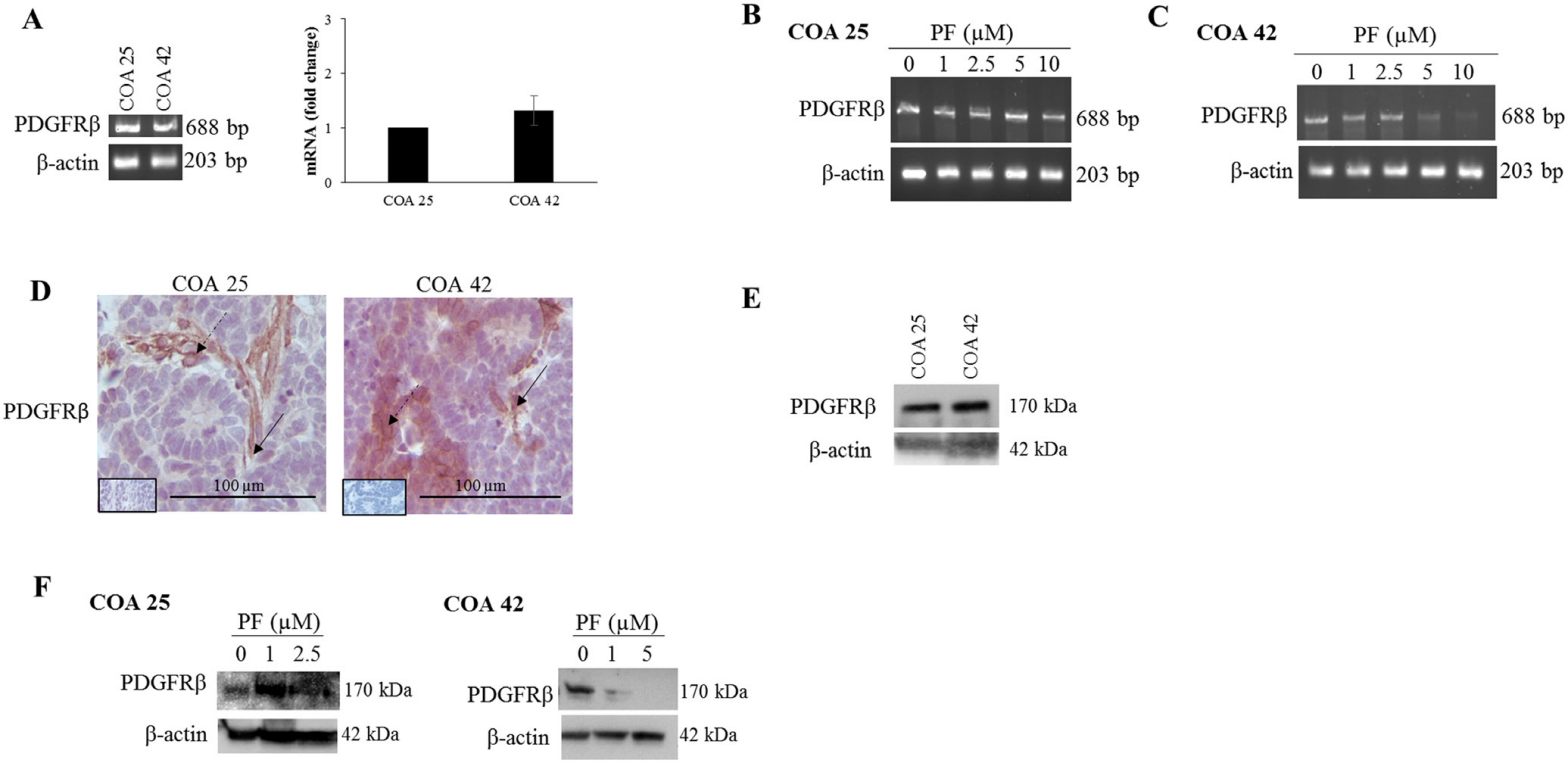
PF-573,228 (PF) inhibition of FAK altered PDGFRβ in COA 25 and COA 42 cells. (**A**) Real-time reverse transcription PCR and gel electrophoresis for PDGFRβ were performed using 0.5 µg of extracted RNA from COA 25 and COA 42 cells. Abundance of PDGFRβ mRNA did not significantly differ between COA 25 and COA 42 cells. (**B**) PCR for PDGFRβ mRNA was performed using 0.5 µg of extracted RNA from COA 25 cells treated with increasing concentrations of PF. Abundance of mRNA for PDGFRβ did not appear significantly different in PF-treated COA 25 cells. (**C**) PCR for PDGFRβ mRNA was performed using 0.5 µg of extracted RNA from COA 42 cells treated with increasing concentrations of PF. Abundance of mRNA for PDGFRβ was significantly decreased in PF-treated COA 42 cells. (**D**) Immunohistochemical staining with antibodies for PDGFRβ was performed on PDXs COA 25 and COA 42. Negative controls were included for each sample (*inserts*). Staining for PDGFRβ was positive and located in tumor cell cytoplasm and membrane (*dashed arrows*) and in tumor stroma (*solid arrows*) of PDXs COA 25 and COA 42. (**E**) Immunoblotting for PDGFRβ was performed on COA 25 and COA 42 cell lysates. PDGFRβ was detected in both PDXs. (**F**) COA 25 and COA 42 cells were treated for 24 hours with increasing concentrations of PF. Cell lysates were harvested and evaluated with immunoblotting for PDGFRβ. Treatment with PF increased expression of PDGFRβ in COA 25 cells while decreasing expression in COA 42 cells.

Of the potential pathways revealed by kinomic profiling, PDGFRβ was chosen for further investigation as its expression is associated with metastasis and poor prognosis in other solid tumors including renal cell carcinoma [30–32]. Using real-time reverse transcription PCR and gel electrophoresis, PDGFRβ mRNA abundance was first examined at baseline and then after FAK inhibition with PF in both PDXs. While mRNA abundance for PDGFRβ appeared to be lower at baseline in COA 25 cells compared to COA 42 cells, this finding was not statistically significant (Figure 4A, *right panel*). Additionally, PDGFRβ mRNA abundance appeared unchanged in COA 25 cells (Figure 4B) but decreased in COA 42 cells (Figure 4C) with PF treatment.

After examining PDGFRβ at the level of mRNA, protein expression was then examined in untreated and PF-treated samples. Immunohistochemical staining for PDGFRβ demonstrated its expression in both PDXs (Figure 4D). Both PDXs also expressed PDGFRβ by immunoblotting of cell lysates (Figure 4E). FAK inhibition with PF increased expression of PDGFRβ in COA 25 cells (Figure 4F, *left panel*) and decreased expression in COA 42 cells (Figure 4F, *right panel*).

### Treatment with sunitinib decreased cell survival and proliferation

**Figure 5:**
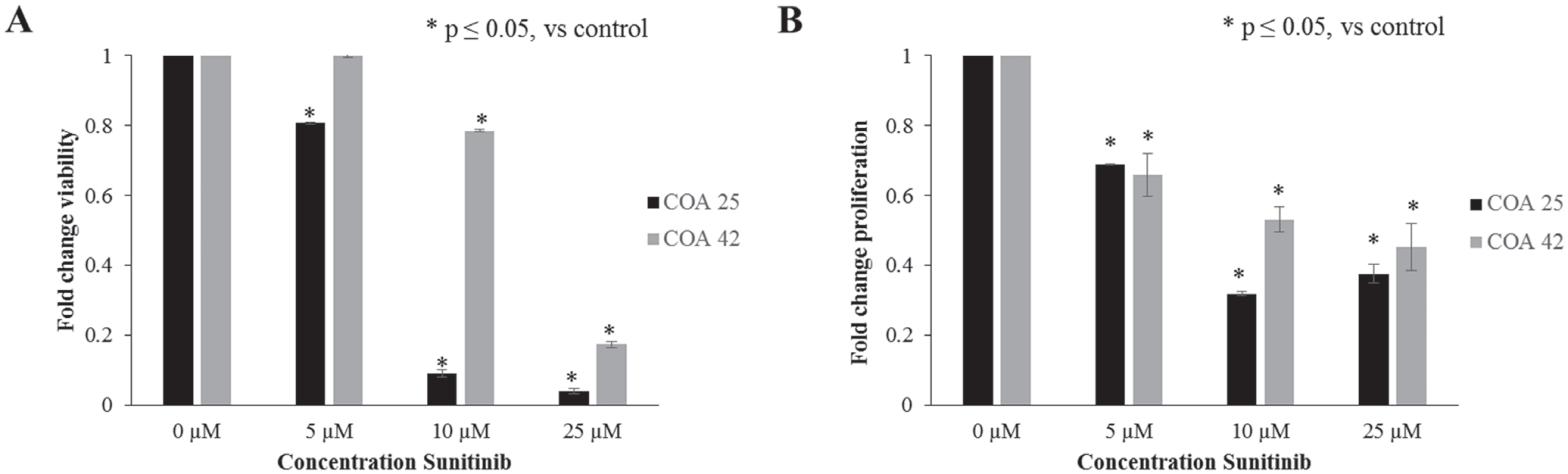
Sunitinib decreased cell survival and proliferation. (**A**) COA 25 and COA 42 cells (6 × 10^4^/well) were treated for 24 hours with increasing concentrations of sunitinib. AlamarBlue^®^ assays were used to assess cell survival. Both COA 25 and COA 42 showed significantly decreased does-dependent cell survival following treatment with sunitinib for 24 hours. (**B**) COA 25 and COA 42 cells (6 × 10^4^/well) were treated for 24 hours with increasing concentrations of sunitinib. CellTiter 96^®^ assays were used to assess cell proliferation. Both COA 25 and COA 42 showed significantly decreased proliferation following treatment with sunitinib for 24 hours. Data represent at least three biologic replicates and are reported as mean ± SEM.

After establishing the presence of PDGFRβ in primary and metastatic WT PDXs and its associated changes with FAK inhibition, further studies were performed to examine the role of PDGFRβ inhibition. Cells were treated with sunitinib at increasing concentrations for 24 hours and survival was evaluated with alamarBlue^®^ assays. Both PDXs, COA 25 and COA 42, demonstrated significantly decreased cell viability following treatment with sunitinib (Figure 5A). The calculated LD_50_ for sunitinib in COA 25 cells was 9.6 µM and in COA 42 cells was 16.7 µM. The LD_50_ for sunitinib in HEK 293 cells was 104.9 µM. Additionally, using CellTiter96^®^ assays, treatment with sunitinib was found to significantly decrease cell proliferation in both COA 25 and COA 42 PDXs (Figure 5B).

### Dual inhibition of FAK and PDGFRβ had a synergistic effect on decreasing cell viability

**Figure 6:**
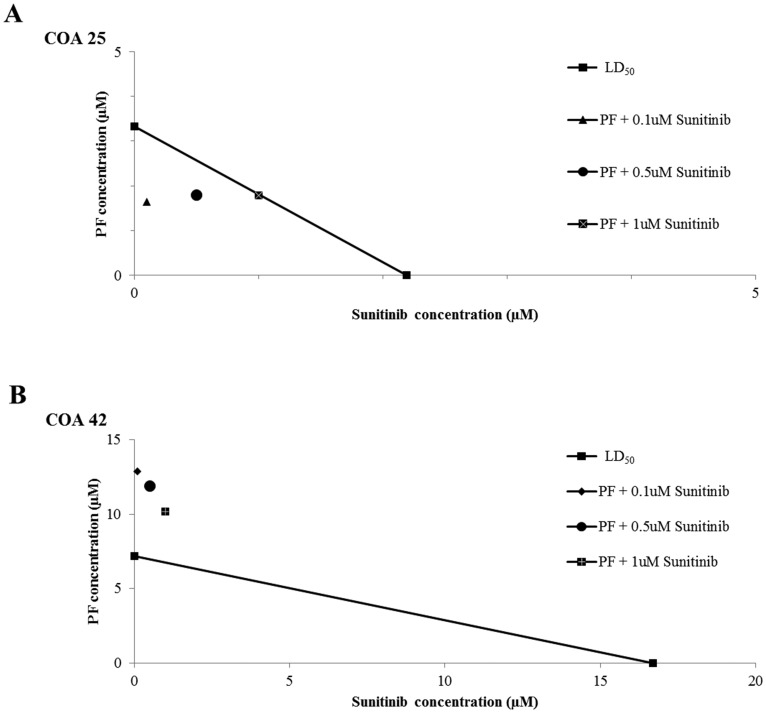
Dual treatment of WT PDX cells with PF-573,228 (PF) and sunitinib had a synergistic effect in decreasing cell viability in COA 25 cells. (**A**) COA 25 cells (6 × 10^4^/well) were treated for 24 hours with increasing concentrations of PF and sunitinib alone or in combination. Cell viability was measured with alamarBlue^®^ assays. The LD_50_ of each drug alone was plotted on the x- and y-axes and connected by a diagonal line. Combination points were plotted. A combination point below the diagonal line indicates synergism. All combination points fell below the line, indicating synergy between the two drugs. (**B**) COA 42 cells (6 × 10^4^/well) were treated for 24 hours with increasing concentrations of PF and sunitinib alone or in combination. Cell viability was measured with alamarBlue^®^ assays. The LD_50_ of each drug alone was plotted on the x- and y-axes and connected by a diagonal line. Combination points were plotted. A combination point below the diagonal line indicates synergism. No combination point fell below the line, indicating a lack of synergy between the two drugs.

To assess the effect of FAK inhibition with PF in combination with sunitinib, cell viability was assessed for COA 25 and COA 42 cells treated with PF alone, sunitinib alone, or a combination of the two drugs for 24 hours. Isobolograms were constructed and CIs calculated (Figure 6; Supplementary Table 2). For COA 25 cells, values fell below the line (CI < 1); therefore, the combination of the two drugs was synergistic such that their effect on cell viability was greater than the effects observed with either drug alone. The combination of PF and sunitinib was not synergistic in the COA 42 metastatic cells (Figure 6B).

## DISCUSSION

Patients with WT who initially present with unfavorable histology with metastatic disease at diagnosis have an overall 4-year survival rate of only 33% [2], highlighting the need for novel therapies for this patient subgroup. FAK and its downstream pathways are involved in a number of tumor-promoting signals [22, 33]. FAK has previously been shown to play a role in tumor cell survival and the development of metastases in pediatric solid tumors such as Ewing sarcoma, renal tumors, neuroblastoma, and hepatoblastoma [20, 21, 28, 34, 35]. FAK has also been shown to be up-regulated in renal cell carcinoma and its expression correlated with poorer patient survival [36, 37]. In this study, we sought to explore the role of FAK inhibition in metastatic WT using a novel PDX model of a primary WT and its matched isogenic metastatic tumor.

After demonstrating the presence of FAK and phosphorylated FAK in both PDXs, we proceeded to examine the effects of FAK inhibition with PF-573,228 and Y15. PF-573,228 works by ATP competitive kinase inhibition of FAK (Y397) while Y15 blocks access to FAK (Y397) in an ATP-independent fashion [38, 39]. With increasing concentrations, these two drugs decreased total FAK expression in both PDXs and these findings have been noted in other cancer types such as pancreatic cancer [40, 41]. Prior pediatric studies utilizing PF and Y15 demonstrated decreased cell viability and proliferation with an increase in apoptotic markers [19, 21, 28, 35, 42]. Our results demonstrated similar findings with significantly decreased cell survival and proliferation with both PF and Y15 in the primary and metastatic PDXs. The decrease in cell viability was thought to be due to an increase in apoptosis as evidenced by an increase in cleaved caspase 3 and cleaved PARP expression. FAK inhibition also resulted in a lack of progression through the cell cycle in these PDX tumor cells.

While FAK inhibition affected both the primary and metastatic PDXs, there were notable differences between the two. First, the LD_50_ for both PF and Y15 were lower for the metastatic compared to the primary PDX. Secondly, while the primary PDX grew in soft agar, a marker of cell invasion, the metastatic PDX cells did not. Finally, FAK inhibition decreased migration and invasion in the primary PDX, but it was unable to do so in the metastatic PDX. Our findings of a less invasive metastatic PDX is similar to a study of orthotopic injections of a primary WT cell line, WT-CLS1, compared to a metastatic WT line, WiT49 [25]. In that study, the primary cell line readily metastasized to the liver and lungs while the metastatic cell line did not. Investigators have hypothesized that in cells of the same tumor type, there may be variable dependence on survival factors leading to different effects with inhibition which may help to explain the differences observed [43].

Kinomic profiling has previously been used in other studies to describe malignancies such as renal cell carcinoma [24]. To further examine differences in pathways involving FAK between primary and metastatic WT, kinomic profiling was performed and demonstrated several differences between the primary and metastatic WT PDX at baseline and after FAK inhibition with PF. We chose to examine PDGFRβ because inhibition of PDGFRβ signaling inhibited tumor growth and degree of lung metastasis in an *in vivo* murine model of renal cell carcinoma [44]. Additionally, PDGFRβ expression has been shown to correlate with poor prognosis in renal cell carcinoma [32].

With regards to WT, while some information is known about PDGFRα, little is known about the expression and role of PDGFRβ. An analysis of 62 pre-treated patient WTs demonstrated that PDGFRα was primarily expressed in epithelial components and its expression correlated with a favorable prognosis [45]. Additionally, mutations in PDGFRα have not been found to play a role in WT [46]. During embryogenic development of the kidney, PDGFRβ is expressed in undifferentiated metanephric blastema, vascular structures, and interstitial cells, and as the glomerular tuft forms, PDGFRβ is primarily expressed within mesangial cells [47]. Studies have shown that high expression of PDGFRβ is predictive of poorer prognosis in renal cell carcinoma [32] but no studies have examined its expression in WT.

In the current study, immunohistochemical staining for PDGFRβ demonstrated its presence in the cell cytoplasm and membrane of both PDXs. This staining pattern was previously shown in localized renal cell carcinoma where its expression correlated with the development of distant metastases. The authors of that study hypothesized that PDGFRβ expression in localized disease may be part of the early events leading to metastasis [32]. PDGFRβ has also been shown to be expressed on stromal cells that support neo-angiogenic vessels in solid tumors, such as small cell lung cancer and prostate cancer [48–50] and our study also demonstrated PDGFRβ in the stroma of both PDXs. Complex interactions between tumor cells and their microenvironment, including the development of a rich vascular blood supply, are required for metastasis [51] and given its location, our results suggest PDGFRβ is involved in these interactions in WT. While kinomic profiling demonstrated PF treatment increased PDGFRβ activity in COA 25 cells, mRNA abundance for PDGFRβ was not affected but immunoblotting revealed increased PDGFRβ expression. Meanwhile, PF treatment decreased mRNA abundance and PDGFRβ expression in COA 42 cells despite having no effect on PDGFRβ activity by kinomic profiling. Discrepancies between gene expression, protein expression, and enzymatic activity of cancer cells have previously been reported [52] and may be due to post-transcriptional, translational, and degradation regulation [24, 53]. Taken together, these findings suggest FAK inhibition may increase PDGFRβ activity as a compensatory mechanism for tumorigenesis in primary WT.

While there are no specific PDGFRβ inhibitors, sunitinib, a small-molecule inhibitor of several receptor tyrosine kinases, was chosen as it has a high specificity for PDGFRβ [54]. Sunitinib was also chosen as it has clinical significance as an FDA-approved treatment for several adult cancers including renal cell carcinoma [55, 56]. Sunitinib’s role in WT has not been examined, but results of the current study suggest that there may be one. Treatment of both primary and metastatic WT PDX cells with sunitinib demonstrated decreased viability and proliferation. Although these findings may be due to off-target effects since sunitinib inhibits a number of other receptor tyrosine kinases, prior studies have demonstrated a lower IC_50_ for PDGRFβ compared to kinases such as VEGFR and c-Kit [57]. Studies have also shown sunitinib targeted down-regulation of PDGFRβ in a variety of solid tumor cell lines both *in vitro* and *in vivo* [48, 49, 58].

Sunitinib’s potential role in WT was also seen in our study by PF and sunitinib acting synergistically to decrease cell survival in primary WT PDX cells. These results are supported by previous studies demonstrating sunitinib in combination with PF-562,271, another small molecule FAK inhibitor, had a greater anti-tumor effect with decreased tumor volume and increased tumor necrosis compared to monotherapy for human hepatocellular carcinoma in a rat xenograft model [59]. Synergism between PF and sunitinib was not seen in the metastatic WT PDX; however, this may be explained by the finding that FAK inhibition with PF decreased PDGFRβ expression (Figure 4F), thereby diminishing the target for sunitinib to act upon. Overall, these findings have potential clinical implications for treating WT as Phase I studies of FAK inhibitors in adults with recurrent or metastatic solid tumors have demonstrated tolerability and stabilization of disease [26, 27]. Furthermore, sunitinib has been shown to be tolerated by children and young adults with recurrent or refractory solid tumors [60–62]. The significantly higher cell survival of HEK 293 cells in our study in comparison to both PDXs after treatment with FAK or PDGFRβ inhibition suggests that normal renal cells do not experience significant toxicity with such inhibition and supports these studies demonstrating tolerability. Finally, FAK inhibitors in combination with other kinase inhibitors, such as trametinib, an inhibitor of the mitogen activated protein kinase pathway, have been shown to be tolerated in young adults [63].

In summary, we reported that FAK was expressed and phosphorylated in both primary and metastatic WT PDXs. FAK inhibition had a significant effect upon the tumor cell survival and motility of both primary and metastatic WT PDXs, more so in the primary. FAK inhibition increased PDGFRβ activity in a primary WT PDX. Using a combination of PF and suninitib, we also demonstrated a synergistic effect on decreasing tumor cell survival in the primary WT PDX. We believe that the data presented provide evidence for further investigation of FAK and PDGFRβ inhibition for the treatment of WT.

## MATERIALS AND METHODS

### Human Wilms tumor xenografts

COA 25 and COA 42 were established from a pediatric patient under informed consent and institutional review board approval (IRB X130627006). The female patient from whom COA 25 and COA 42 were derived was 3 years old at diagnosis with Stage III, favorable histology WT. COA 25 was established at initial biopsy of the primary renal tumor, prior to any treatment. The child received chemotherapy per the Children’s Oncology Group protocol AREN0532 with regimen DD-4A. At week 22 of therapy, a hepatic relapse was found on routine imaging and a hepatic resection was performed leading to the establishment of COA 42.

The xenografts were maintained by serial transplantation in athymic nude mice. The University of Alabama at Birmingham (UAB) Institutional Animal Care and Use Committee approved the uses of all animal subjects and all animal experiments described below (IACUC 09064). Authentication of the human cell lines was determined by short tandem repeat proﬁling performed by the UAB Heﬂin Center for Genomic Science.

### Tumor disaggregation and cell culture

PDX tumors were measured weekly and aseptically harvested when measurements reached at least 1500 mm^3^ in size. Tumors were disaggregated to produce single cell suspensions using a gentleMACS Dissociator (Miltenyi Biotec, Auburn, CA) per manufacturer’s standard protocol. Cells were washed with Roswell Park Memorial Institute (RPMI) medium (Thermo Fisher Scientific, Rockford, IL). Pellets of COA 25 and COA 42 cells were maintained in NeuroBasal medium (Life Technologies, Carlsbad, CA) prepared with ﬁbroblast growth factor–b and epidermal growth factor (Miltenyi) at 10 ng/mL, with 2% B-27 supplement without vitamin A (Life Technologies), 2% N2 supplement (Life Technologies), 2 mM L-glutamine, amphotericin B (250 mg/mL) (Corning, Lowell, MA), and gentamicin (50 mg/mL) (Corning). All tumor cells were maintained under standard conditions at 37°C and 5% CO_2_ in their respective media. All experiments were performed with cells harvested directly from the animal and not passed through cell culture conditions.

The human embryonic kidney cell line HEK 293 (CRL-1573, American Type Culture Collection, ATCC, Manassas VA) was utilized as a normal cell control. Cells were maintained in Dulbecco’s Modified Eagle Medium (Mediatech, Wembley, WA) containing 10% heat-activated fetal bovine serum (Atlanta Biologicals, Flowery Branch, GA), 0.05% Trypsin-EDTA 1X (Gibco, Carlsbad, CA), and 1% penicillin-streptomycin (Corning).

### Antibodies and reagents

Monoclonal rabbit anti-phospho-FAK (Tyr 397, 700255) was obtained from Invitrogen (Rockford, IL). Polyclonal rabbit anti-FAK (C-20) was obtained from Santa Cruz Biotechnology (Dallas, TX). Polyclonal rabbit anti-cleaved PARP (AB3565) was obtained from EMD Millipore (Billerica, MA). Polyclonal rabbit anti-cleaved caspase 3 (9661S) was obtained from Cell Signaling Technology (Danvers, MA). Monoclonal rabbit anti-PDGFRβ (32570) was obtained from Abcam (Cambridge, MA). Monoclonal mouse anti-β-actin (A1978) was obtained from Sigma Aldrich (St. Louis, MO). The small molecule FAK inhibitor, PF-573,228 (PF;C_22_H_20_F_3_N_5_O_3_S), was obtained from Santa Cruz. A second small molecule FAK inhibitor, 1,2,4,5-benzenetetraamine tetrahydrochloride (Y15; C_6_H_10_N_4_·4ClH), was obtained from Sigma Aldrich. Sunitinib (C_22_H_27_FN_4_O_2_·C_4_H_6_O_5_), a receptor tyrosine kinase inhibitor known to target PDGFRβ, was obtained from Selleckchem (Houston, TX).

### Immunohistochemistry

Formalin-fixed, paraffin-embedded human WT specimens and xenograft tumor specimens were cut into 5 µm sections, baked at 70°C for 1 hour, deparaffinized, rehydrated, and steamed. Sections were quenched with 3% hydrogen peroxide and blocked with blocking buffer [bovine serum albumin (BSA), powdered milk, Triton X-100, phosphate buffered saline (PBS)] for 30 minutes at 4°C. The monoclonal primary mouse anti-FAK (EMD Millipore 05-537) and rabbit anti-phospho-FAK (Invitrogen 700255) antibodies were added at a 1:500 dilution and incubated overnight at 4°C. After washing with PBS, the secondary antibody (R.T.U. Biotinylated anti-rabbit/mouse IgG, Vector Laboratories, Burlingame, CA) was added for 1 hour at 22°C. The staining reactions were developed with VECTASTAIN Elite ABC kit (PK-7200, Vector Laboratories) and DAB (10X, 1856090, Thermo Fisher Scientific). Slides were counterstained with hematoxylin. Negative controls [mouse IgG (1 mg/mL, Invitrogen) or rabbit IgG (1 mg/mL, EMD Millipore)] were included with each run. Similar methods were used for the monoclonal primary rabbit anti-PDGFRβ antibody (MA5-14851, Thermo Fisher Scientific) added at a 1:200 dilution and incubated overnight at 4°C.

### Immunoblotting

Cells or homogenized tumor specimens were washed with cold 1× PBS and lysed on ice for 1 hour in radioimmunoprecipitation assay buffer (RIPA) [10 mmol/L Tris base pH 7.2, 150 mmol/L NaCl, 1% Na-deoxycholate, 1% Triton X-100, 0.1% sodium dodecyl sulfate (SDS)] supplemented with protease inhibitors, phosphatase inhibitors, and phenylmethanesulfonylfluoride (Sigma Aldrich). Lysates were cleared by centrifugation at 14000 rpm for 1 hr at 4°C. Pierce BCA Protein Assay Reagent (Thermo Fisher Scientific) was used to determine protein concentrations. Boiled samples were then separated by electrophoresis on SDS polyacrylamide gels. Antibodies were used according to manufacturers’ recommendations. The expected size of target proteins was confirmed with molecular weight markers (Precision Plus Protein Kaleidoscope Standards, Bio-Rad, Hercules, CA). Immunoblots were developed with Luminate Classico or Crescendo ECL (EMD Millipore). Stripping solution (Bio-Rad) at 37°C for 15 minutes was used to strip blots which were then re-probed with selected antibodies. Equal protein loading was confirmed with immunoblotting with antibody to β-actin. Densitometry relative to β-actin using Image J software Ver 1.49 (imagej.nih.gov) was also performed.

### Cell viability and proliferation assays

Cell viability was measured with alamarBlue^®^ assays. Cells were plated (6 × 10^4^ cells/well) in 96-well culture plates and treated with PF, Y15 or sunitinib at increasing concentrations for 24 hours. AlamarBlue^®^ dye (10 µL, Thermo Fisher Scientific) was added and the absorbance at 570 nm and 600 nm was measured using a microplate reader (BioTek Gen5, Ver 3.02, BioTek Instruments, Winooski, Vermont). Viability was reported as a mean fold change ± SEM of at least three biologic replicates.

Cell proliferation was measured with CellTiter 96^®^ assays. Cells were plated (1.5 × 10^4^ cells/well) on 96-well cultures plates and treated with PF or Y15 at increasing concentrations for 24 hours. For 24-hour treatment with increasing concentrations of sunitinib, 6 × 10^4^ cells/well on 96-well culture plates were used. CellTiter 96 Aqueous One Solution^®^ (10 µL, Promega, Madison, WI) was added and absorbance at 490 nm was measured. Proliferation was reported as a mean fold change ± SEM of at least three biologic replicates.

### Cellular motility assays

Twelve-well culture plates (Corning) with 5 µm micropore inserts were used. To measure cellular migration, the top side of the insert was coated with Matrigel™ (1 mg/mL; BD Biosciences) overnight at 37°C. Cells (5 × 10^5^) pre-treated with PF or Y15 were plated into the top well. To measure cellular invasion, in addition to coating the top side of the insert with Matrigel™, the bottom side was coated with laminin (10 µg/mL, Cultrex, Cambridge, MA). After 24 hours, inserts were fixed with 3% paraformaldehyde and stained with crystal violet. Pictures of the inserts (×10) were taken using the image software SPOT Basic 5.2 (Diagnostic Instruments Inc., Heights, MI). The percentage of area invaded or migrated was analyzed using Image J and reported as a mean fold change ± SEM of at least three biologic replicates.

### Attachment-independent growth assay

Soft agar assay was used to measure attachment-independent growth. A base layer of complete culture media in 2% noble agar was made in 60-mm culture dishes. Cells (3 × 10^5^) were plated in the top layer composed of the same culture media and agar mixture. Dishes were treated with PF or Y15 and retreated every 4 days. After incubation at 37°C for 8 weeks, colonies were stained with crystal violet, imaged using Gel Dock Imager (Bio-Rad, Hercules, CA) and quantified using Image J.

### Cell-cycle analysis

Cells (1 × 10^6^) pre-treated with increasing concentrations of PF or Y15 for 24 hours were treated with accutase, washed with PBS, and then fixed in 100% ethanol overnight. Cells were then washed with PBS, stained with a solution of 0.1% Triton X/PBS (Thermo Fisher Scientific), RNAse A (20 mg/mL; Invitrogen), and propidium iodide (1 mg/mL; Invitrogen) for 1 hour at 4°C and analyzed with fluorescence activated cell sorting (FACS) using a FACSCalibur^TM^ Flow Cytometer (Becton Dickinson Biosciences, San Jose, CA). Negative controls were included in each flow cytometry run. ModFit LT software (Verity Software House Inc., Topsham, ME) was used to interpret data.

### Kinomic profiling

Kinomic profiling was used as a hypothesis-generating tool to examine potential therapeutic targets upstream and downstream of FAK. Cells (5 × 10^6^) pre-treated with PF (0, 2.5 µM) for 24 hours were washed with PBS. Protein from cell lysates were then combined with kinase buffer, ATP, and fluorescently labeled antibodies and loaded into a phosphotyrosine kinase or serine-threonine kinase PamChip^®^ per the UAB Kinome Core protocol [23]. Phosphopeptide substrate analysis with the PamStation^®^12 kinomics workstation (PamGene^®^ International), PamChip^®^ protocol using Evolve2 Software, and BioNavigator v. 6.0 were used to analyze kinases upstream and downstream of FAK.

### Real-time reverse transcription PCR

RNA was extracted from untreated cells and cells pre-treated with PF for two-step real-time reverse transcription PCR using manufacturer’s recommendations for an RNeasy Mini Kit (Qiagen, Valencia, CA). A Take3™ micro-volume plate and microplate reader (BioTek Gen5, Ver 3.02, BioTek Instruments, Winooski, Vermont) were used to measure 0.5 µg of RNA per sample. RNA was then reverse transcribed using a iScript™ cDNA Synthesis Kit (Bio-Rad). cDNA was then amplified using a T100 Thermal Cycler (Bio-Rad) with samples kept for 5 minutes at 25°C followed by 30 minutes at 42°C and 5 minutes at 85°C, with an infinite hold at 4°C. The DNA samples were then used for real-time PCR using a Bio-Rad SsoAdvanced™ Universal SYBR^®^ Green Supermix probe and primers for PDGFRβ and β-actin (Invitrogen). Sequences for forward and reverse PDGFRβ primers, respectively, were: 5′-AATGTCTCCAGCACCTTCGT-3′ and 5′-AGCGGATGTGGTAAGGCATA-3′. Sequences for forward and reverse β-actin primers, respectively, were: 5′-GGCATCCTCACCCTGAAGT-3′ and 5′-GGGGTGTTGAAGGTCTCA-3′. Real-time PCR was performed on CFX Connect™ Real-Time PCR Detection System and analyzed using CFX Manager (Bio-Rad). Each treatment group was performed in quadruplicate and threshold cycle (C_T_) values for the PDGFRβ gene were averaged for each group and normalized to expression levels of β-actin. The delta-delta C_T_ value (x) was then calculated by taking the treated delta C_T_ value and subtracting the control sample delta C_T_ value. Results were then reported as a fold change by calculating 2^^-x^.

### Gel electrophoresis

Gel electrophoresis for PDGFRβ was performed using 10 µL of amplified PCR samples as described above. Gels were 2% agar made from Bio-Rad Certified Molecular Biology Agarose and 1× Tris base, acetic acid, and EDTA buffer. SYBR^®^ Safe DNA Gel Stain (Invitrogen) was used to probe for bands. The expected size of target bands was confirmed with Thermo Fisher Scientific GeneRuler™ Express DNA Ladder. Equal loading was confirmed with β-actin. Immunoblots were developed using ChemiDoc™ MP Imaging System and Image Lab™ 5.0 (Bio-Rad).

### *In vitro* combination studies

Cell viability was measured using alamarBlue^®^ assays as above. Cells (6 × 10^4^) were treated for 24 hours with PF alone, sunitinib alone, or a combination of PF and sunitinib at varying concentrations. Combination indices (CIs) were calculated and isobolograms constructed using the Chou-Talalay method [64].

### Data analysis

Experiments were repeated with at least three biologic replicates and data reported as mean ± SEM. Results were compared with student’s *t*-test using Microsoft Excel and *p* ≤ 0.05 was considered significant.

## SUPPLEMENTARY MATERIALS



## Abbreviations

WT: Wilms tumor
FAK: Focal adhesion kinase
PDX: Patient-derived xenograft
PDGFRβ: Platelet-derived growth factor receptor beta
PF: PF-573,228
Y15: 1,2,4,5-benzenetetraamine tetrahydrochloride
