# Supplementary material for: The effects of focal adhesion kinase and platelet-derived growth factor receptor beta inhibition in a patient-derived xenograft model of primary and metastatic Wilms tumor
Source: Oncotarget. 2019 Sep 17;10(53):5534–48. doi: 10.18632/oncotarget.27165 (PMC6756857; doi:10.18632/oncotarget.27165)
Supplement: Supplementary file 1 [file oncotarget-10-5534-s001.pdf]

The effects of focal adhesion kinase and platelet-derived growth factor receptor beta inhibition in a patient-derived xenograft model of primary and metastatic Wilms tumor

SUPPLEMENTARY MATERIALS

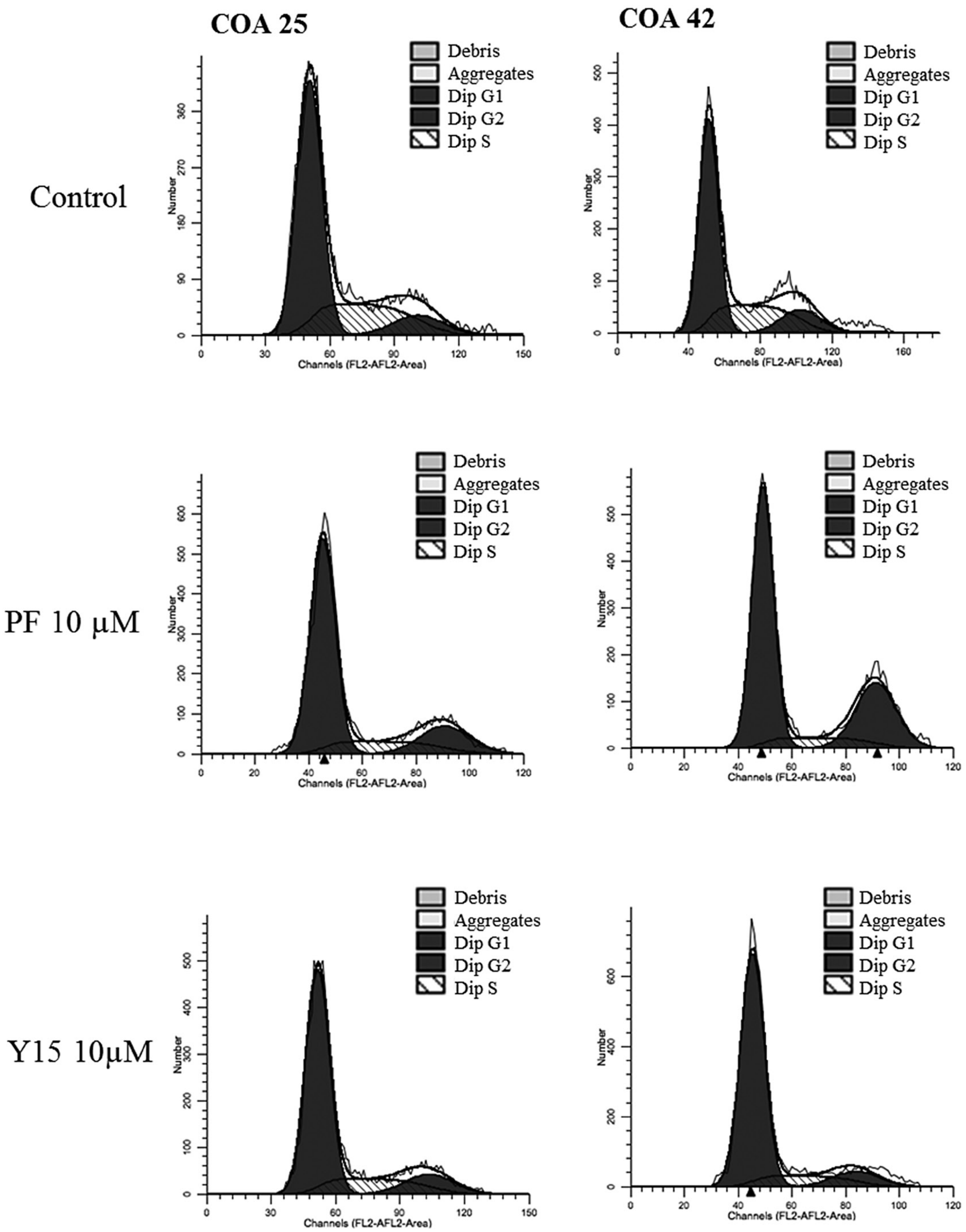

Supplementary Figure 1: PF-573,228 (PF) and 1,2,4,5-benzenetetraamine tetrahydrochloride (Y15) inhibition of FAK decreased cell cycle progression. COA 25 and COA 42 cells (1×10^6) were treated for 24 hours with PF or Y15. Representative histograms of cell cycle as measured by FACS of COA 25 and COA 42 demonstrated an increased number of cells in G_1 and decreased number of cells in S phase with treatment with PF and Y15 (10 μM) compared to controls.

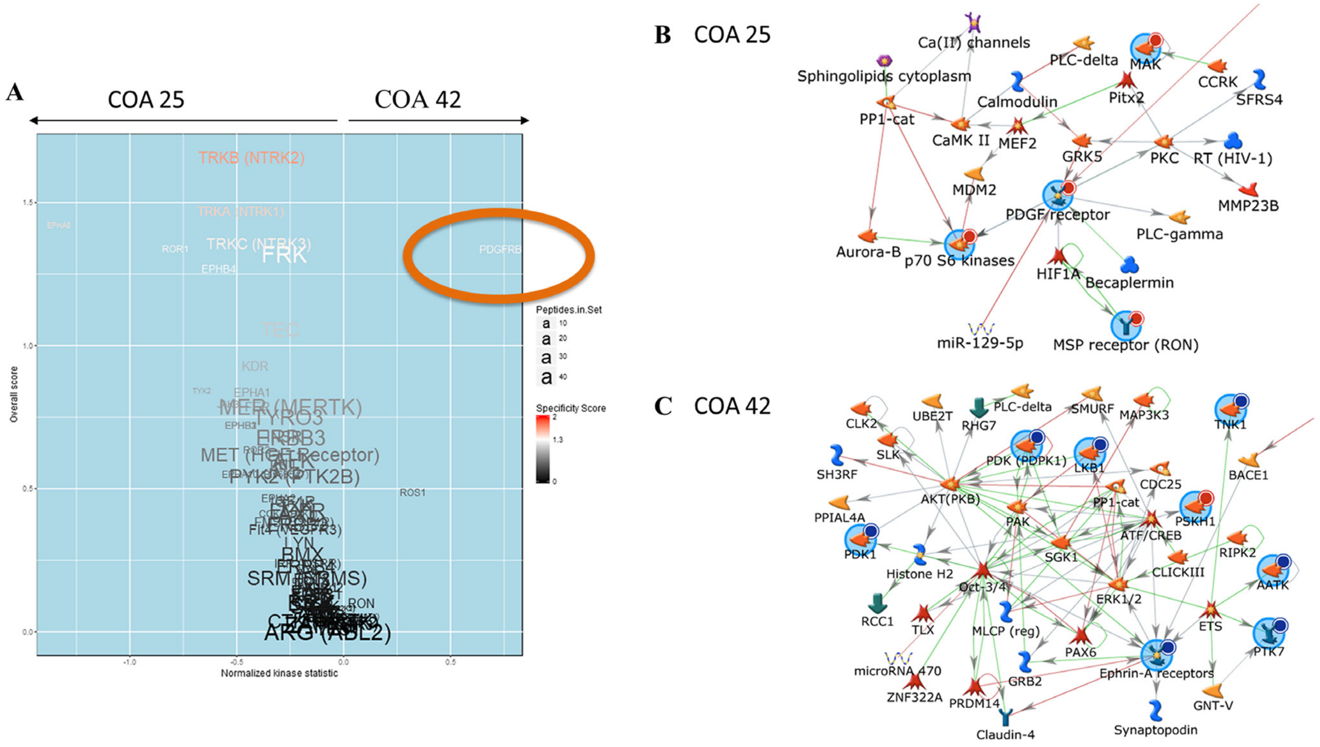

Supplementary Figure 2: Tyrosine and serine/threonine kinomic analysis of COA 25 and COA 42 cells. (**A**) Kinase scoring and specificity of COA 25 and COA 42 cells. Cells (5×10^6) were lysed and protein was quantified and loaded into a PamChip® per the UAB Kinome Core protocol. COA 25 had increased EPHA8 and ROR1 and decreased PDGFRβ activity relative to COA 42. (**B**) Network modeling of PF-573,228 (PF) treated COA 25 cells. Cells (5×10^6) were treated with PF (2.5 μM) for 24 hours prior to cell lysis, protein quantification, and analysis by PamChip® per the UAB Kinome Core protocol. Red circles in the upper right of a node indicate a PF-increased kinase. Treatment with PF increased PDGFRβ, RON, P70S6KB, and MAK activity in COA 25 cells. (**C**) Network modeling of PF-573,228 (PF) treated COA 42 cells. Cells (5×10^6) were treated with PF (2.5 μM) for 24 hours prior to cell lysis, protein quantification, and analysis by PamChip® per the UAB Kinome Core protocol. Red circles in the upper right of a node indicate a PF-increased kinase while blue circles indicate a PF-decreased kinase. Treatment with PF decreased TNK1, LMR1, CCK4, EPHA5, PDK1, SGK196, LKB1 and increased PSKH1 activity in COA 42 cells.

Supplementary Table 1: Cell cycle analysis of COA 25 and COA 42 treated with PF or Y15

	G_1	G_2	S
**COA 25**			
Control	62.95% ± 1.19%	9.76% ± 0.84%	27.30% ± 0.71%
PF (10 μM)	69.35% ± 1.75%*	15.37% ± 2.31%*	15.28% ± 0.58%*
Y15 (10 μM)	70.91% ± 0.26%*	11.56% ± 0.24%	17.53% ± 0.10%*
**COA 42**			
Control	59.12% ± 1.47%	13.31% ± 0.68%	27.57% ± 2.12%
PF (10 μM)	61.73% ± 0.18%	27.58% ± 1.01%*	10.69% ± 0.85%*
Y15 (10 μM)	78.10% ± 0.16%*	9.08% ± 0.17%*	12.81% ± 0.29%*

PF-573,228 (PF) and 1,2,4,5-benzenetetraamine tetrahydrochloride (Y15) inhibition of FAK decreased cell cycle progression. COA 25 and COA 42 cells (1×10^6) were treated for 24 hours with PF or Y15. Cell cycle analysis demonstrated a significantly increased percentage of cells in G_1 and a decreased percentage in S phase for COA 25 cells treated with PF (10 μM), indicating a lack of progression through the cell cycle. Analysis also demonstrated similar results for both COA 25 and COA 42 cells following treatment with Y15 (10 μM). Cell cycle analysis was repeated with at least three biologic replicates and reported as mean percentage in phase ± SEM.

$^*p \leq 0.05$, control vs. treatment.

Supplementary Table 2: Combination indices for COA 25 and COA 42 treated with sunitinib and PF

COA 25	LD (%)	Sunitinib (μM)	PF (μM)	CI
	50	0.1	1.65	0.541
	50	0.5	1.81	0.772
	50	1	1.79	0.995
**COA 42**	**LD (%)**	**Sunitinib (μM)**	**PF (μM)**	**CI**
	50	0.1	12.82	1.787
	50	0.5	11.92	1.686
	50	1	10.13	1.467

Dual treatment of WT PDX cells with PF-573,228 (PF) and sunitinib had a synergistic effect in decreasing cell viability in COA 25 cells. COA 25 and COA 42 cells (6×10^4/well) were treated for 24 hours with increasing concentrations of PF and sunitinib alone or in combination. Cell viability was measured with alamarBlue® assays. Combination indices (CIs) were calculated with a CI < 1 indicating synergism. All CIs for COA 25 cells were less than 1, indicating synergy between the two drugs.

LD: lethal dose. CI: combination index.
